# Time to Meaningful Clinical Response Across Approved and Emerging Therapies for Antihistamine-Refractory Chronic Spontaneous Urticaria: A Network Meta-Analysis

**DOI:** 10.3390/jcm15124622

**Published:** 2026-06-14

**Authors:** Sarayu Balachandar, Dylan R. Clapp, Alan B. Fleischer

**Affiliations:** 1 University of Cincinnati College of Medicine, Cincinnati, OH 45267, USA; clappdr@mail.uc.edu; 2 Department of Dermatology, University of Cincinnati College of Medicine, Cincinnati, OH 45267, USA

**Keywords:** chronic spontaneous urticaria, network meta-analysis, UAS7, urticaria activity score

## Abstract

**Background/Objectives**: Several novel biologics and small-molecule therapies have emerged for the treatment of antihistamine-refractory chronic spontaneous urticaria (CSU), yet no study has directly compared their speed of response. This study aims to provide indirect evidence on the relative time to meaningful clinical response across approved and investigational therapies using a Bayesian network meta-analysis. **Methods**: Phase 2 and phase 3 randomized controlled trials reporting UAS7 scores in a graphical format for antihistamine-refractory CSU were included. The primary outcome was the mean time in weeks to minimal clinically important difference (MCID), defined as a UAS7 reduction of 10 points. Data were extracted using WebPlotDigitizer (v4.7) and analyzed via Bayesian random-effects network meta-analysis in MetaInsight (v6.4.0), with placebo as the reference node. **Results**: All drugs except rilzabrutinib 400 mg daily demonstrated faster mean time to MCID than placebo. Fenebrutinib had the fastest mean time to MCID (0.67–0.76 weeks), and tezepelumab the slowest (5.41–5.65 weeks). Only omalizumab 300 mg every 4 weeks, dupilumab 300 mg every 2 weeks, and ligelizumab 72 mg and 120 mg every 4 weeks achieved statistically significant reductions compared with placebo. All treatments had wide credible intervals reflecting limited direct comparisons. **Conclusions**: This is the first network meta-analysis comparing time to meaningful symptom control across therapies for antihistamine-refractory CSU. Omalizumab, dupilumab, and ligelizumab demonstrated statistically significant reductions in time to MCID compared with placebo. Head-to-head trials with standardized outcome reporting would enable more definitive comparative conclusions.

## 1. Introduction

Chronic spontaneous urticaria (CSU) is an inflammatory skin condition characterized by mast-cell-mediated pruritic wheals, angioedema, or both that persist for more than 6 weeks. Symptoms persist beyond 1 year in most patients and for 3 years or longer in up to two-thirds of patients, with a substantial impact on quality of life comparable to that seen in psoriasis or atopic dermatitis. CSU is increasingly recognized as an autoimmune condition, driven by distinct endotypes involving IgE and IgG autoantibodies that account for mast-cell activation in more than half of patients [1].

Second-generation H1-antihistamines are the current first-line therapy for CSU. Escalation of therapy is recommended in patients with inadequate control, defined as persistent symptoms despite up to four times the standard dose of antihistamines for 2–4 weeks [2]. Although standard-dose second-generation H1-antihistamines achieve at least a partial response in approximately 39% of patients, fewer than 10% of patients achieve complete disease control at standard doses, and only about 5% do so even with high-dose regimens [1,2]. Over the last decade, several novel therapies with distinct mechanisms of action have been developed, including anti-IgE (omalizumab, ligelizumab), anti–IL-4/IL-13 (dupilumab), Bruton tyrosine kinase (BTK) inhibitors (remibrutinib, fenebrutinib, rilzabrutinib), and thymic stromal lymphopoietin (TSLP) inhibitors (tezepelumab) [3]. Of these, omalizumab, dupilumab, and remibrutinib are currently approved by the Food and Drug Administration (FDA) for antihistamine-refractory CSU [4].

The Urticaria Activity Score over 7 days (UAS7) is the standard criterion for assessing disease activity in CSU clinical trials and routine care [5], with a change of approximately 10 points considered as the threshold for a clinically meaningful improvement [6]. Given the limited number of head-to-head randomized controlled trials directly comparing therapies for antihistamine-refractory CSU [3], this study aims to provide indirect evidence on the relative speed of response among emerging agents using a Bayesian network meta-analysis. We hypothesize that understanding time to meaningful improvement may not only guide shared decision-making between patients and healthcare providers but also inform expectations for adherence, cost-effectiveness, and the design of future clinical trials. 

## 2. Materials and Methods

A systematic search of the MEDLINE database was conducted to identify randomized controlled trials evaluating biologics and small-molecule therapies for antihistamine-refractory chronic spontaneous urticaria. Both FDA-approved and investigational therapies were included. Search terms included “chronic spontaneous urticaria,” “randomized controlled trial,” and “UAS7”. Only phase 2 or phase 3 trials presenting UAS7 scores over time (weeks) in graphical format were included, as this enabled precise estimation of the time required to reach a minimal clinically important difference (MCID). Other study inclusion criteria included a minimum of 10 patients per treatment arm and a comparison arm, both of which were necessary to conduct a meta-analysis. For drugs without phase 3 trial data, all doses evaluated across phase 2 trials were included in the analysis. For drugs with phase 3 trial data, only the doses carried forward into phase 3 were selected from phase 2 trials for comparison. For FDA-approved drugs, only the approved dose(s) were included. Authors independently screened each study to confirm eligibility. Since the analysis is based entirely on previously published, publicly available data, no institutional review board approval or patient consent was necessary.

The primary outcome for this analysis was the average time to MCID in weeks, defined as a UAS7 score reduction of 10 points [6]. WebPlotDigitizer (v4.7) was used to extract data from the graphical UAS7 plots in each included study, with weeks on the x-axis and mean UAS7 scores on the y-axis. The time at which the mean UAS7 score reached a 10-point reduction from baseline, corresponding to the MCID, was determined for each treatment arm and placebo arm. For the placebo arms of each trial, if the predetermined MCID was never reached, the last recorded follow-up was selected as an imputed mean time. Given the varying methodologies of all studies involved and differences in data reporting, common standard deviations (SD) were not available for each of the studies. To account for this, a common coefficient of variation (CV) was selected and set to 0.5 [7]. This coefficient was then multiplied across each study’s mean time-to-MCID (SD = CV × Mean) to give an approximation of standard deviation for each study. 

**Figure 1:**
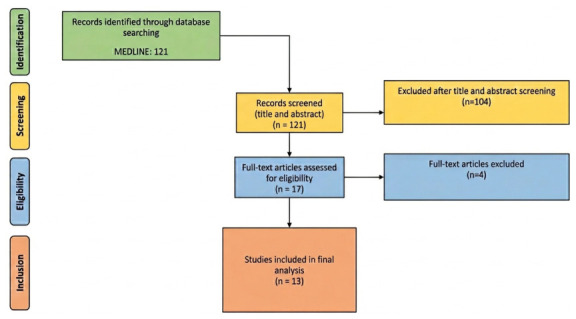
PRISMA flow diagram depicting the systematic search and study selection process.

All extracted data were uploaded into MetaInsight (v6.4.0), and a Bayesian random-effects network meta-analysis (NMA) was run through Markov Chain Monte Carlo simulations, allowing for generation and sampling of many random distributions of the included data. Additional details regarding the Bayesian NMA model specifications, MCMC settings, prior distributions, and model fit assessment are provided in the Supplementary Materials. When conducting the NMA, MetaInsight accounts for the mean and imputed SD, along with the sample sizes of each study, to perform a weighted analysis of each study in the network. In this analysis, each treatment arm functions as a node within a connected network. Placebo arms were included to ensure network connectivity and served as the reference node for all treatment comparisons [7,8]. The power of the Bayesian NMA is in the modeling of both between-study and within-study variance. Each selected study varies in terms of trial design, drug class, dosing regimen, and study population. The Bayesian NMA accounts for study heterogeneity and generates a distribution of relative treatment effects that reflects the uncertainty in the included data [9]. This review adheres to the PRISMA guidelines as stated in the 2020 PRISMA statement: guidelines for systematic reviews [10]. The study selection process is summarized in Figure 1.

## 3. Results

Thirteen randomized controlled trials out of seventeen met the inclusion criteria and were included in the meta-analysis (Table 1), with 32 unique arms (trial drug vs. placebo) comprising the network comparison. The network was placebo-anchored, with all trials using placebo as the primary comparison, and only two (PEARL-1 and PEARL-2) including alternative medications as secondary comparisons. 

**Figure 2:**
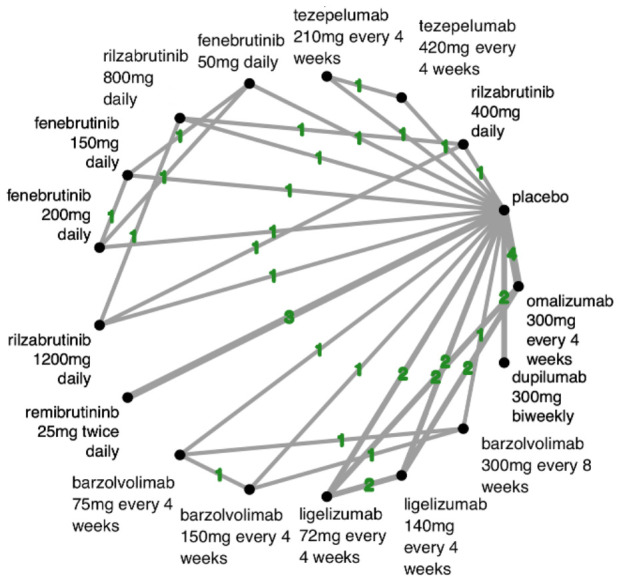
Network map showing comparisons between trial drugs and placebo. The number on each line represents the number of trials that were compared between the two connected nodes.

Figure 2 shows the network with the included trial arms. Each point represents a trial node, with the lines connecting nodes indicating where comparisons were made. The numbers over each line indicate how many studies are included in that given comparison. This plot shows that each study is consistently compared to the placebo, with limited direct drug-to-drug comparisons. It does not give any information on the strength of the connection or the significance of MCID changes. The between-study standard deviation was 7.28 (95% CI 4.21–13.14), with higher numbers indicating more variance and heterogeneity between included studies.

**Figure 3:**
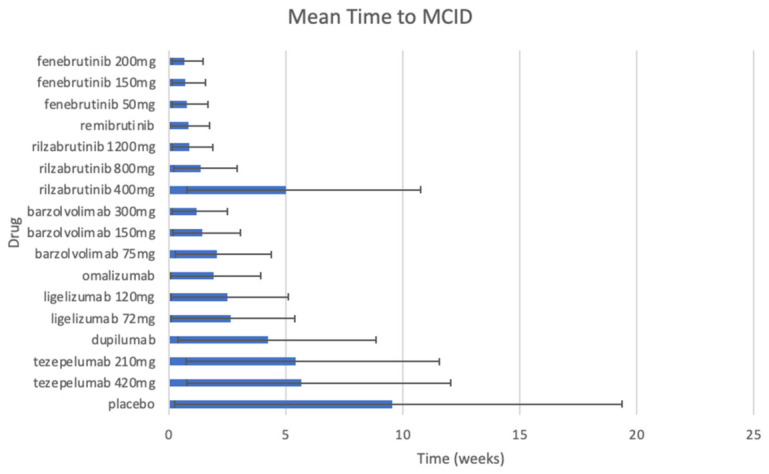
Mean (95% CI) time in weeks to reach MCID for each drug and dose included in this study.

The mean time to MCID for each drug–dose combination included in the analysis is shown in Figure 3. All investigated drugs had a faster mean time to MCID than placebo before significance testing. The drug with the fastest reported mean time to MCID was fenebrutinib (0.67, 0.71, and 0.76 weeks at each trial dose), though it did not reach significance and thus conclusions cannot be drawn regarding its effectiveness. The drug with the slowest overall mean time to MCID was tezepelumab (5.41 and 5.65 weeks). However, the credible intervals for all drugs overlapped, likely due to limited sample inclusion.

**Figure 4:**
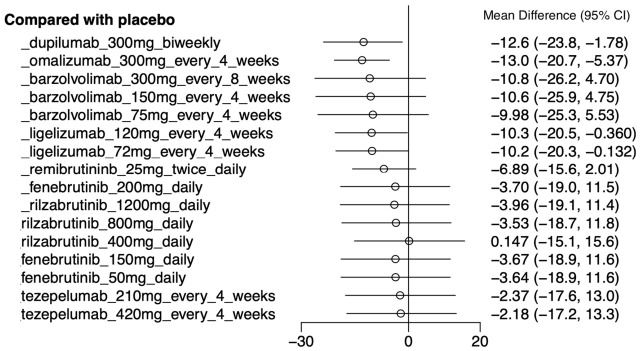
Forest plot showing the mean difference in time to reach MCID for each included drug and dose compared to placebo.

In all evaluated treatments, only three drugs reached statistically significant MCID’s: omalizumab 300 mg given every 4 weeks (mean difference −13.0, 95% CI [−20.7 to −5.57]), dupilumab 300 mg given every 2 weeks (mean difference −12.6, 95% CI [−23.8 to −1.78]), and ligelizumab. Both doses included in the ligelizumab trial (72 mg every 4 weeks and 120 mg every 4 weeks) met significance (mean difference −10.2, 95% CI [−20.3 to −0.132], and mean difference −10.3, 95% CI [−20.5 to −0.360] respectively). All other treatments, except rilzabrutinib 400 mg taken daily, were associated with mean reductions in MCID compared with placebo. Notably, the other FDA-approved drug, remibrutinib, did not reach significance when compared with placebo, potentially due to the limited studies available for inclusion. Mean changes in MCID with associated credible intervals are shown in Figure 4.

Notably, all treatments included in the analysis had very wide credible intervals despite consistent and potentially relevant decreases in mean time to MCID. This reflects the substantial uncertainty arising from both inter- and intra-study variance, with limited direct comparisons. Indirect comparisons via placebo nodes introduce imprecision and contribute to wide credible intervals that could be obscuring more clinically relevant results.

## 4. Discussion

CSU has been characterized as an autoimmune disease by several authors, based on two recognized endotypes. Type I autoimmune (autoallergic) CSU is mediated by IgE autoantibodies directed against self-antigens such as thyroid peroxidase, while type IIb autoimmune CSU involves mast-cell-activating IgG autoantibodies targeting either IgE or its high-affinity receptor (FcεRI). Together, these mechanisms account for mast-cell activation in more than half of patients. When activated, mast cells release bioactive mediators, including histamine and leukotrienes that bind to histamine receptors, causing vasodilation and fluid extravasation from blood vessels, resulting in the characteristic wheals and itch [25,26].

Second-generation H1-antihistamines work by blocking peripheral H1 receptors, preventing histamine from binding and thereby reducing vasodilation and pruritus. Their major limitation is that they target only one downstream mediator while leaving upstream mast-cell activation intact. They do not address the autoimmune mechanisms driving mast-cell degranulation, nor do they prevent the release of other inflammatory mediators, such as leukotrienes and cytokines. Additionally, dose escalation increases somnolence (9% vs. 5% at standard doses), although second-generation agents cross the blood–brain barrier less than first-generation antihistamines [27].

This is the first study to compare the time to achieving meaningful symptom control across available therapeutic options for the management of antihistamine-refractory CSU using a network meta-analysis framework. As with all placebo-anchored network meta-analyses, findings reflect the inherent limitations of indirect comparison, and conclusions about the relative speed of response should be considered hypothesis-generating pending direct head-to-head trial data.

Omalizumab at 300 mg administered every 4 weeks and dupilumab at 300 mg every 2 weeks achieved statistically significant reductions in time to MCID compared with placebo, consistent with their established roles as second-line therapies for CSU. Omalizumab reduces free IgE levels and downregulates FcεRI expression on mast cells and basophils, preventing the crosslinking of IgE-FcεRI complexes that trigger mast-cell activation [1]. In clinical practice, omalizumab achieves complete disease control in approximately 72% of patients and is supported by the largest evidence base among CSU biologics, including three phase 3 trials (ASTERIA I, ASTERIA II, GLACIAL) [14,15,22]. Dupilumab blocks IL-4 and IL-13 signaling via the IL-4 receptor alpha subunit, inhibiting type 2 inflammation through a pathway distinct from omalizumab [10]. In the CUPID phase 3 trials, dupilumab demonstrated efficacy regardless of baseline IgE levels, suggesting it may benefit patients who do not achieve complete control with omalizumab [11]. Regarding safety, omalizumab’s long-term profile is well-established, with injection-site reactions being the most common adverse event [14,15], whereas dupilumab carries a similar safety profile but with a more limited duration of follow-up in CSU specifically [11]. Notably, while both agents achieved significant reductions in time to MCID in this analysis, their mechanisms target different points in the inflammatory cascade, which may have implications for differential response across patient endotypes [25,26]. While time to meaningful response is an important consideration, it must be weighed alongside each therapy’s adverse effect profile and contraindications when guiding individualized treatment decisions.

Ligelizumab, a next-generation high-affinity anti-IgE monoclonal antibody, also achieved statistically significant reductions in time to MCID at both the 72 mg and 120 mg doses every 4 weeks. While ligelizumab demonstrated superiority over placebo in the PEARL-1 and PEARL-2 phase 3 trials, it failed to show superiority over omalizumab on the primary endpoint, leading to discontinuation of its development for CSU [13]. Despite binding IgE with higher affinity than omalizumab, ligelizumab did not translate this pharmacologic advantage into a faster or greater clinical response, underscoring that affinity alone may not predict therapeutic superiority [13,21]. Qualitative mechanistic differences, including weaker inhibition of IgE:CD23 interactions and inability to dissociate IgE already bound to FcεRI, may offset ligelizumab’s affinity advantage [28]. These findings suggest that the efficacy of anti-IgE therapies is influenced by a broader mechanistic profile that extends beyond binding affinity, and that statistical significance versus placebo does not necessarily translate to clinical superiority over existing therapies.

Remibrutinib has recently received FDA approval for the treatment of antihistamine-refractory CSU, further expanding treatment options. As a selective Bruton tyrosine kinase (BTK) inhibitor, remibrutinib acts upstream of mast-cell degranulation by blocking FcεRI-mediated signaling, offering an oral alternative to injectable biologics. In the phase 3 REMIX-1 and REMIX-2 trials, remibrutinib 25 mg twice daily demonstrated significant improvements in UAS7 and complete response rates compared with placebo. Overall, it was well tolerated, with headache and nasopharyngitis among the most common adverse events [17]. It did not reach significance in this analysis, though this should not be interpreted as the absence of efficacy, as it demonstrated meaningful reductions versus placebo in its pivotal trial [16]. The non-significant result most likely reflects the structural limitations of this analysis, including limited direct comparisons and wide credible intervals, rather than true null effects. The remaining investigational agents trended toward benefit but did not reach significance, except for rilzabrutinib 400 mg daily, which showed no advantage over placebo, possibly reflecting a sub-therapeutic dose.

Despite these constraints, this analysis offers several methodological strengths. This study benefits from the inclusion of both FDA-approved and investigational therapies, providing a broad comparative view of the current and emerging treatment landscape for antihistamine-refractory CSU. Additionally, the Bayesian network meta-analysis framework is particularly well-suited to this context, as it enables indirect comparisons across trials that differ in design and population. By anchoring all comparisons to a shared placebo node, the model explicitly accounts for between-study heterogeneity, an important consideration given the variability across the included trials.

Several limitations should be considered. Many included therapies are newly developed agents for which clinical trial data remain limited, resulting in a sparse evidence network with relatively few direct head-to-head comparisons. Several agents with available trial data were not included, such as lirentelimab, because they did not meet eligibility criteria. Substantial variability across trials, including differences in study populations, dosing regimens, and outcome reporting, introduces heterogeneity that contributes to wide credible intervals. Because time to MCID was not routinely reported as a trial endpoint, outcome data had to be digitally extracted from published graphical representations. For example, the ASTERIA II phase 3 trial for omalizumab was excluded entirely as it did not report UAS7 as a mean change from baseline [29]. This highlights the need for standardized outcome reporting frameworks in CSU trials, comparable to initiatives such as Harmonizing Outcome Measures for Eczema (HOME) in atopic dermatitis, which mandate consistent reporting of core outcome measures to allow for cross-trial comparisons [30]. Furthermore, standard deviations for time-to-MCID were not consistently available and were therefore imputed, which may affect the precision of the estimate. Finally, limited sample sizes for certain treatment arms and reliance on indirect comparisons increase uncertainty in relative treatment estimates. Future trials with standardized time-to-response reporting will be beneficial in drawing more definitive comparative conclusions across the expanding therapeutic landscape of antihistamine-refractory CSU [3].

## 5. Conclusions

In conclusion, this network meta-analysis represents the first indirect comparison of time to meaningful symptom control across approved and investigational therapies for antihistamine-refractory CSU. Omalizumab, dupilumab, and ligelizumab demonstrated statistically significant reductions in time to MCID compared with placebo, highlighting the therapeutic potential of targeted IgE and type 2 inflammatory pathway blockade for rapid symptom control. The wide credible intervals across all treatments emphasize the need for adequately powered head-to-head trials with a standardization of reported outcomes to enable more definitive comparisons across the expanding CSU-treatment landscape.

## Figures and Tables

**Table 1:** Studies meeting the inclusion criteria for the UAS7 analysis.

Authors	Article Title	Met Criteria for Analysis
Maurer et al. [11]	Dupilumab in patients with chronic spontaneous urticaria (LIBERTY-CSU CUPID): Two randomized, double-blind, placebo-controlled, phase 3 trials. (CUPID A)	Yes
Maurer et al. [11]	Dupilumab in patients with chronic spontaneous urticaria (LIBERTY-CSU CUPID): Two randomized, double-blind, placebo-controlled, phase 3 trials. (CUPID B)	Yes
Giménez-Arnau et al. [12]	Rilzabrutinib in Antihistamine-Refractory Chronic Spontaneous Urticaria: The RILECSU Phase 2 Randomized Clinical Trial.	Yes
Maurer et al. [13]	Efficacy and safety of ligelizumab in adults and adolescents with chronic spontaneous urticaria: results of two phase 3 randomized controlled trials (PEARL-1 and PEARL-2).	Yes
Maurer et al. [13]	Efficacy and safety of ligelizumab in adults and adolescents with chronic spontaneous urticaria: results of two phase 3 randomized controlled trials (PEARL-1 and PEARL-2).	Yes
Saini et al. [14]	Efficacy and Safety of Omalizumab in Patients With Chronic Idiopathic/Spontaneous Urticaria Who Remain Symptomatic on H1 Antihistamines: A Randomized, Placebo-Controlled Study (ASTERIA I).	Yes
Kaplan et al. [15]	Omalizumab in patients with symptomatic chronic idiopathic/spontaneous urticaria despite standard combination therapy (GLACIAL).	Yes
Maurer et al. [16]	Remibrutinib, a novel BTK inhibitor, demonstrates promising efficacy and safety in chronic spontaneous urticaria.	Yes
Metz et al. [17]	Remibrutinib in Chronic Spontaneous Urticaria (REMIX-1).	Yes
Metz et al. [17]	Remibrutinib in Chronic Spontaneous Urticaria (REMIX-2).	Yes
McLaren et al. [18]	Tezepelumab for the treatment of chronic spontaneous urticaria: Results of the phase 2b INCEPTION study.	Yes
Metz et al. [19]	Fenebrutinib in H1 antihistamine-refractory chronic spontaneous urticaria: a randomized phase 2 trial.	Yes
Metz et al. [20]	Randomized dose-finding study of anti-KIT barzolvolimab in patients with chronic spontaneous urticaria.	Yes
Maurer et al. [21]	Ligelizumab for Chronic Spontaneous Urticaria.	No
Maurer et al. [22]	Omalizumab for the Treatment of Chronic Idiopathic or Spontaneous Urticaria (ASTERIA II).	No
Saini et al. [23]	A randomized, placebo-controlled, dose-ranging study of single-dose omalizumab in patients with H1-antihistamine-refractory chronic idiopathic urticaria.	No
Altrichter et al. [24]	An open-label, proof-of-concept study of lirentelimab for antihistamine-resistant chronic spontaneous and inducible urticaria.	No

## Data Availability

Data can be provided on request to the authors.

## Supplementary Materials

The following supporting information can be downloaded at: https://www.mdpi.com/article/10.3390/jcm15124622/s1, Supplemental Methods: Detailed Bayesian network meta-analysis model specifications, including MCMC settings, prior distributions, and model fit assessment. Supplementary Figure S1. Leverage plot for model fit assessment of the Bayesian network meta-analysis.
