# Supplementary material for: Time to Meaningful Clinical Response Across Approved and Emerging Therapies for Antihistamine-Refractory Chronic Spontaneous Urticaria: A Network Meta-Analysis
Source: J Clin Med. 2026 Jun 14;15(12):4622. doi: 10.3390/jcm15124622 (PMC13301777; doi:10.3390/jcm15124622)
Supplement: Supplementary file 1 [file jcm-15-04622-s001.zip › jcm-4297598-supplementary.pdf]

Supplemental Methods: Detailed Bayesian network meta-analysis model specifications, including MCMC settings, prior distributions, and model fit assessment.

The following provides additional detail on the Bayesian NMA model specifications. Using MetaInsight (v6.4.0), a Bayesian NMA was conducted through Markov Chain Monte Carlo (MCMC) simulations. Characteristics of the performed MCMC analysis include a burn-in run of 5000 simulations, which acts as a primer for the dataset and helps reduce variability introduced by early simulations. Following burn-in, a total of 20,000 sample iterations were conducted to generate the final sample dataset. A noninformative/vague prior distribution was used for the MCMC, with a mean of 0 and variance of 112,694.5. The included heterogeneity prior was 0 to 22.4. A leverage plot was generated to assess model fit and is provided in Supplementary Figure S1.

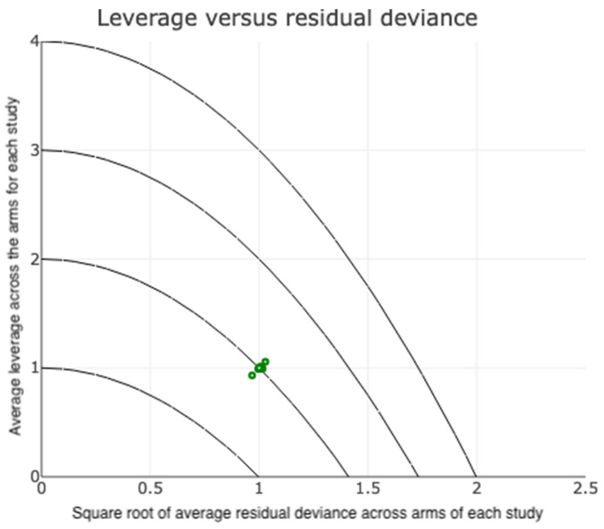

Supplementary Figure S1. Leverage plot for model fit assessment of the Bayesian network meta-analysis.

This shows the leverage of each study versus the residual deviance of each study. Leverage is the posterior mean of residual deviance minus the deviance of the posterior mean of the fitted values. The lines indicate study arm fit, with points lying off the lines contributing to poorer fit of the NMA model. The further off the line each node is indicates the worse fit to model for that study. Most studies included in this model have relatively good fit, the 2 studies with the worst fit are Phase 2 remibrutinib study (below the line), and the REMIX-2 trial (above the line). Posterior density plots and Gelman convergence assessments can be made available upon request. All parameters are consistent with default Bayesian NMA MCMC settings in MetaInsight and were not adjusted in any way prior to analysis.
